# Variation in species composition and infection rates of *Anopheles* mosquitoes at different altitudinal transects, and the risk of malaria in the highland of Dirashe Woreda, south Ethiopia

**DOI:** 10.1186/s13071-017-2288-0

**Published:** 2017-07-19

**Authors:** Taye Yohannes Daygena, Fekadu Massebo, Bernt Lindtjørn

**Affiliations:** 1 Department of Biology, Arba Minch University, Arba Minch, Ethiopia; 2 Dirashe Woreda Health Office, Malaria and Other Vector-Borne Diseases Control Unit, Gidole, Ethiopia; 3 Centre for International Health, University of Bergen, Bergen, Norway

**Keywords:** Altitudinal variation, *Anopheles arabiensis*, *Anopheles demeilloni*, Dirashe Woreda

## Abstract

**Background:**

The transmission of malaria is heterogeneous, and varies due to altitude. The information on whether the transmission of malaria is indigenous or imported to highland areas is scarce. Therefore, this study aimed to assess the species composition and infection rates of *Anopheles* at different altitudinal transects, and the risk of malaria if any in the highland of Dirashe Woreda, South Ethiopia.

**Methods:**

This study was conducted in Gato (low altitude; average elevation of 1273 m), Onota (mid-altitude; average elevation of 1707 m) and Layignaw-Arguba (high altitude; average elevation of 2337 m) from August 2015 to April 2016. *Anopheles* mosquitoes were sampled using Centers for Disease Control and Prevention (CDC) light traps from thirty houses (ten houses from each village). The circum-sporozoite proteins (CSPs) rate and entomological inoculation rate (EIR) of *Anopheles* mosquitoes were estimated. For the epidemiological survey, malaria cases were collected from laboratory registration books of selected health facilities from (August 2015-April 2016). A cross-sectional survey was done to collect data on malaria vector control activities in each village (August-September 2015).

**Results:**

One thousand two hundred sixty-eight *Anopheles* mosquitoes comprising *Anopheles arabiensis*, *An. demeilloni*, *An. cinereus*, *An. pharoensis*, *An. funestus-*group, *An. pretoriensis*, *An. christyi*, *An. ardensis* and *An. tenebrosus* were identified in the study area. *Anopheles arabiensis* was the dominant species in Gato, whereas *An. demeilloni* was the dominant species in Layignaw-Arguba. Five mosquitoes, three *An. arabiensis* from Gato and two *An. demeilloni* from Layignaw-Arguba, were positive for *Plasmodium falciparum* CSPs. *Plasmodium falciparum* CSP rate was 0.4% (95% CI: 0.08–1.15) for *An. arabiensis* in Gato, and it was 0.64% (95% CI: 0.08–2.3) for *An. demeilloni* from Layignaw-Arguba. The *P. falciparum* EIR of *An. arabiensis* was 8.6 (95% CI: 2.4–33.4) infectious bites/person/nine-months in Gato. *Plasmodium falciparum* parasite was dominant in Gato (88%) and Onota (57.5%), whereas in Layignaw-Arguba *P. vivax* (59.4%) occurred most frequently. Increased malaria cases were observed in children age 5–14 years in Gato (*P* < 0.05), whereas in Onota and Layignaw-Arguba there was no statistically significant difference in malaria cases among the age groups. Households owning at least one long lasting insecticidal net were 92.7% in the study area, and 77.6% slept under the net during the preceding night of the survey. About 64.4% of the households in Gato were protected by the indoor residual spray. However, the spraying was done when the density of local malaria vectors was low.

**Conclusion:**

Incrimination of *Plasmodium* CSP positive *Anopheles* species and the presence of malaria in children under five years in high altitude Layignaw-Arguba may justify the existence of indigenous malaria transmission and the need for effective malaria control. Further investigation and confirmation using more sensitive molecular techniques are however needed to consider *An. demeilloni* as a proven vector of malaria in Ethiopia.

## Background

Malaria is a major public health problem of the world with the highest disease burden in sub-Saharan Africa [1]. In Ethiopia, malaria is the leading vector-borne disease and was among the ten leading causes of sickness and deaths in children less than five years of age. The epidemiological pattern of malaria is mostly unstable and seasonal, but its intensity differs from place to place due to altitudinal and climatic variations [2].

The areas with altitude 2000 m above sea level (masl) have been considered as malaria-free, but currently areas above 2400 masl experience local malaria transmission [3, 4]. The expansion of malaria transmission to highland areas has been influenced by the change in climatic conditions [5]. The increasing temperature may shorten the developmental time of the malaria parasite within the mosquito vector and hasten the development of immature stages of *Anopheles* mosquitoes [6, 7]. Moreover, the population in areas with unstable malaria transmission is non-immune and experiences malaria epidemics [8] including the highlands of Ethiopia [9]. Travelling from the highlands to the lowlands for daily activities increases the risk of malaria infection in highland residents [10]. Therefore, the geographical expansion of malaria to the highlands could be a serious public health concern because most of the population lives in the highlands of the country.

In Ethiopia, highlands ranging from an altitude of 1500–2500 m experienced a huge malaria epidemic in 1958 [11]. Also, *Anopheles* species such as *An. arabiensis* (the principal malaria vector), *An. christyi*, *An. demeilloni* and *An. coustani* have been documented in highland areas [3, 12, 13]. Gone et al. [13] have reported *Anopheles* species from lowland and highland of Dirashe. *Anopheles arabiensis*, *An. funestus*, *An. demeilloni* and *An. pharoensis* were documented in low altitude, *An. demeilloni* and *An. cinereus* at mid altitude, and *An. demeilloni* from the high altitude of Dirashe. However, no adequate entomological and parasitological studies were conducted in the area to determine whether the transmission of malaria is indigenous or imported to the highlands. In the previous study conducted by Gone et al. [13], few mosquitoes were collected, and none of them was positive for *Plasmodium* parasites, and therefore, the existing evidence may not be sufficient to conclude the presence of local malaria transmission in the highlands of the study area. Hence, the present study aimed to investigate variation in species composition, sporozoite infection rates of *Anopheles* mosquitoes, and estimate the risk of malaria at different altitudinal transects of Dirashe Woreda in south Ethiopia using circum-sporozoite proteins (CSPs), entomological inoculation rates (EIR), and malaria parasite prevalence.

## Methods

### Study area

**Fig. 1:**
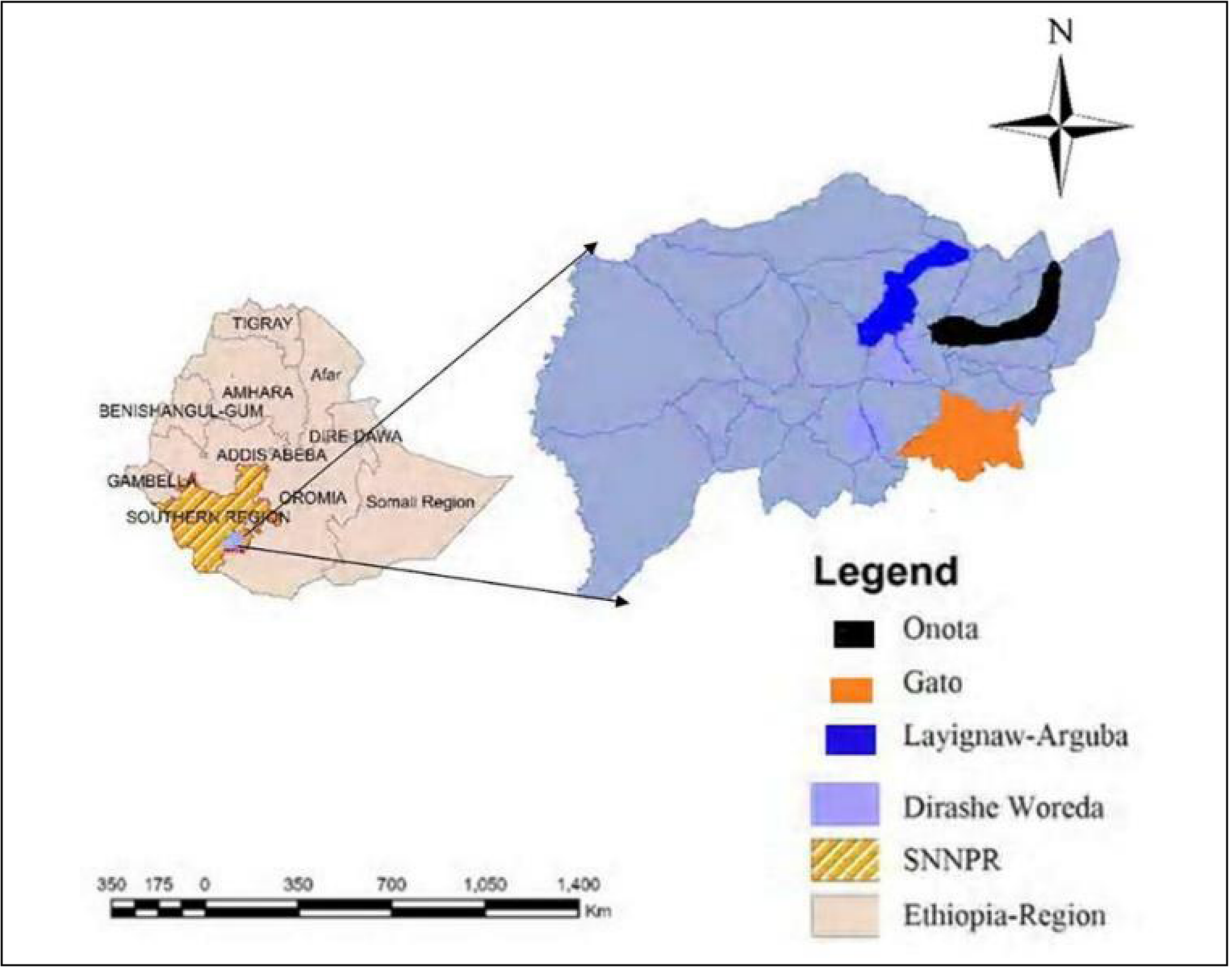
Map of the study areas in Dirashe Woreda, south Ethiopia

This study was conducted in three *kebeles* (villages) at different altitudinal transects in Dirashe Woreda (district) south Ethiopia. These were Gato (low altitude), Onota (middle altitude) and Layignaw-Arguba (high altitude) (Fig. 1). The altitude of Gato is between 1248 and 1298 masl. The other village Onota is 9 km away from Gato and is located at an altitude between 1573 and 1842 masl. Layignaw-Arguba is a highland village with altitude ranging from 2060 to 2614 masl. The soil is fertile, and farming is the main mode of living for the local people. The main crops are maize, sorghum and *teff* (*Eragrostis tef*). Gato is one of the productive villages of the area using irrigation water from the River Yanda which crosses the village from West to East. Onota has varying topography with steep slopes and some plateau in the middle. Surface water is scarce in Onota. On the other hand, Layignaw-Arguba has several swampy areas, many springs, and ponds with a variable topography, vegetation cover and land use.

The annual rainfall ranges between 600 and 1600 mm, and the annual temperature ranges between 15.1 and to 27.5 °C. There is a bimodal rainy season with the long rainy months from March to June and the short rainy months from September to October (unpublished district agricultural office data, 2014). Health services are provided by one primary hospital, four health centres, fourteen health posts and thirty-two private health facilities. The total population of the district is estimated to be 130,276 (63,785 males and 66,491 females). The population of Gato is 12,327. Onota has a population of 6381, whereas the population of Layignaw-Arguba is 6569. The total number of households in the three villages is 5159; Gato has 2516, Onota 1302 and Layignaw-Arguba 1341 (unpublished district health office data of 2015).

### Entomological study designs

A longitudinal entomological study was conducted from August 2015 to April 2016. Adult *Anopheles* mosquitoes were collected twice a month using Centers for Disease Control and Prevention (CDC) light traps. Ten houses were selected randomly from each of the three villages. After obtaining consent from the head of the households, CDC light traps were installed in the sleeping places of the selected houses (from 18:00 to 06:00 h) at about 1.5 m above the floor near the foot end of the person sleeping under the insecticide untreated bednet [14]. A total of 60 CDC light trap-nights per month were conducted. The same households were surveyed throughout the study. The live mosquitoes were sucked by aspirator from collection bags and killed by freezing. Female *Anopheles* mosquitoes were then identified morphologically to species under a stereomicroscope using a morphological identification key [15]. The abdominal status of female *Anopheles* mosquitoes was determined under the microscope into unfed, freshly-fed, half-gravid and gravid. Finally, each female *Anopheles* mosquito was preserved individually in Eppendorf tube containing silica gel for detection of CSPs using enzyme-linked immunosorbent assay (ELISA) [16].

### Processing female *Anopheles* mosquitoes for CSPs detection

The two dominant species, *An. gambiae* (presumably *An. arabiensis*) and *An*. *demeilloni* were analysed for CSP detection using ELISA [16]. The head and thorax of dried females were separated carefully from the abdomen and tested for *P. falciparum* and *P. vivax_*210 CSPs in Arba Minch University Medical Entomology Laboratory.

### Retrospective malaria data collection

The total number of outpatient attendant cases suspected of having malaria was confirmed by microscopic or rapid diagnostic test (RDT) in the selected health centres and health posts. The selection of the health facilities was not random but was based on where most people go for health care. Two health centres and four health posts were used for the survey. Malaria positive cases were categorised using standard age groups for malaria as < 1 year, 1–4 years, 5–14 years and 15+. All microscopic or RDT positive malaria cases were identified and classified into *P. falciparum, P. vivax* and mixed infections.

### Survey of vector control interventions

The sample size required for a cross-sectional survey to assess the vector control intervention coverage and use rate was determined using Epi Info™ (http://www.cdc.gov/epiinfo). The total number of households in the three villages was 5139. To determine the sample size of the survey, 50% malaria control intervention coverage, the margin of error of 4% and 95% confidence level (α = 5%) was considered. Therefore, the total sample size calculated was 538 households. Thus, the sample size of each village was calculated proportionally based on their total number of households. Hence, the number of households needed for the survey in Gato was 262, and it was 136 in Onota and 140 in Layignaw-Arguba.

The households included in the study were selected by systematic random sampling technique. The list of households of the three villages was used as sampling frame with the assumption of similar access to vector control interventions. The first household was selected randomly by lottery method, and every K^th^ household was included in the study. K was calculated by the formula

$$ \mathrm{K}=\frac{N}{n} $$

where K is the gap between every household, N is the total number of households in the study village, and n is the sample size calculated. Since the sample was allocated proportionally, K was the same for each of the study villages.

House to house surveys were conducted by trained data collectors using structured and pre-tested questionnaires. After obtaining consent, the data collector interviewed the household head. The information collected includes malaria prevention and control strategies such as having bednets, the number of bednets, registering if anyone slept under the bednet the night before the survey and insecticide spray status in the last 12 months. The household occupants who lived in the study areas for more than six months were included in the study. If the household’s occupants refused to participate, the household next to it was included in the survey.

### Outcome variables

The primary outcome variables were *Anopheles* mosquito species composition, sporozoite rates and EIR along with the altitudinal transects. Other primary outcomes include the number of malaria cases and *Plasmodium* species at different altitudinal gradients. The secondary outcome variables were the number of households owning LLINs or sprayed, and the number of households using the LLINs the night before the survey in the study villages.

### Data analysis

Data were entered and analysed by using IBM® SPSS® Statistics version 20 (Armonk, New York: IBM Corporation). Analysis of variance was used to compare the density of *Anopheles* mosquitoes and malaria cases at different altitudinal transects of the district. The sporozoite rate was determined for CDC light trap catches. For CDC based EIR, the conversion factor of 1.91 was used [17]. Thus, the standard EIR was calculated as 1.91 multiplied by (number of sporozoite positive ELISAs/number of mosquitoes tested) × (number of mosquitoes collected by CDC/number of CDC catches) [17]. The alternative method was also used to calculate the 95% confidence interval of EIR. The number of malaria cases was estimated as the number of cases per total number of persons suspected and tested for malaria. The difference in bednet use rates in different altitudinal transects and malaria cases in different age groups were compared using the Chi-square test.

## Results

### *Anopheles* species composition

A total of 1268 adult female *Anopheles* mosquitoes comprising nine species were collected. Of these, seven species were found in Gato, three species in Onota and four species in Layignaw-Arguba. *Anopheles gambiae* (*s.l.*) (*An. arabiensis*) was the predominant species (59.5%; 755/1268), followed by *An. demeilloni* (27.4%; 347/1268). The other species were *An. cinereus*, *An. pharoensis*, *An. funestus* group, *An. pretoriensis*, *An. christyi*, *An. ardensis* and *An. tenebrosus* (Table 1).

**Table 1:** *Anopheles* species composition of indoor adult collection using CDC light traps in three study villages of Dirashe Woreda, south Ethiopia (August 2015-April 2016)

*Anopheles* species	Study villages			
	Gato *n* (%)	Onota *n* (%)	Layignaw-Arguba n (%)	Total (%)
*An. arabiensis*	754 (91.1)	1 (3.1)	0 (0)	755 (59.5)
*An. demeilloni*	17 (2.1)	19 (59.4)	311 (76.2)	347 (27.4)
*An. cinereus*	0 (0)	12 (37.5)	90 (22.1)	102 (8.0)
*An. funestus* group	22 (2.7)	0 (0)	0 (0)	22 (1.7)
*An. pharoensis*	22 (2.7)	0 (0)	0 (0)	22 (1.7)
*An. christyi*	0 (0)	0 (0)	6 (1.5)	6 (0.5)
*An. pretoriensis*	7 (0.8)	0 (0)	1 (0.2)	8 (0.6)
*An. ardensis*	3 (0.4)	0 (0)	0 (0)	3 (0.2)
*An. tenebrosus*	3 (0.4)	0 (0)	0 (0)	3 (0.2)
Total	828	32	408	1268

The density of *Anopheles* mosquitoes at different altitudinal transects of the district varied significantly (*F* = 53.7, *df* = 2, *P* < 0.001). *Anopheles arabiensis* was the most common species (91.1%; 754/828) in Gato while *An. demeilloni* was the most prevalent species in Onota (59.4%; 19/32), and in Layignaw-Arguba (76.2%; 311/408). No *An. arabiensis* were collected in Layignaw-Arguba and this species was among the rare species in Onota. *Anopheles christyi* was collected in Layignaw-Arguba only.

**Fig. 2:**
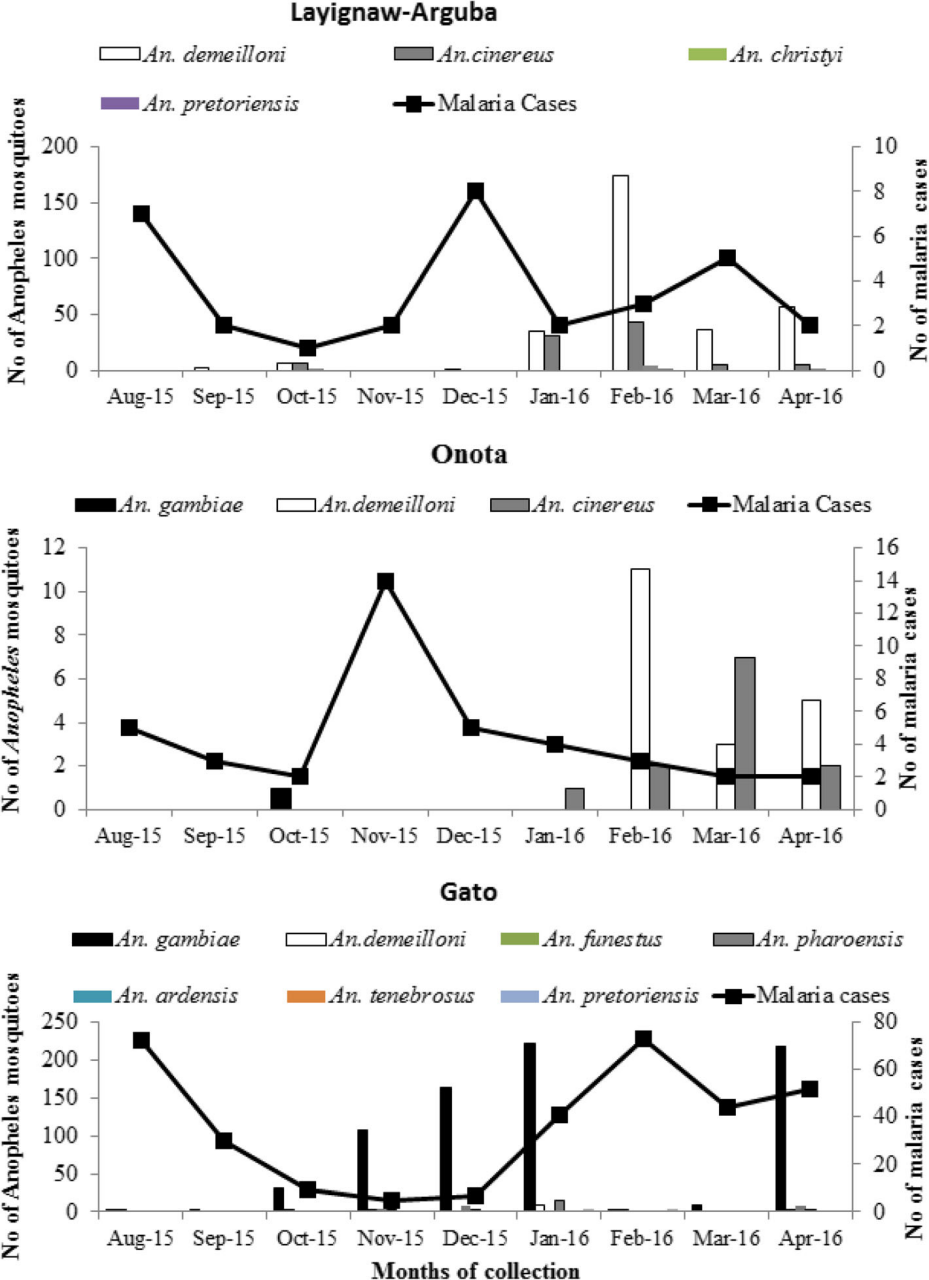
Monthly catches of *Anopheles* mosquitoes and malaria cases trend in the three study villages of Dirashe Woreda, south Ethiopia (August 2015-April 2016)

The abundance of mosquitoes showed seasonal variations (Fig. 2). The monthly density of *Anopheles* mosquitoes varied significantly (*F* = 10.7, *df* = 8, *P* < 0.001). *Anopheles* mosquitoes were collected mainly from November to January, and in April in Gato, from February to April in Onota, and from January to April in Layignaw-Arguba.

### Abdominal status of female *Anopheles* mosquitoes

Of 1268 adult female *Anopheles* mosquitoes collected by CDC light traps, the majority (56.9%; 721) were unfed, and 467 (36.8%) were freshly-fed. Only a small proportion of *Anopheles* mosquitoes were half-gravid and gravid. In Gato, of 828 collected adult *Anopheles* females*,* 499 (60.3%) were unfed and 265 (32.0%) were freshly-fed. In Onota 71.9% (23 of the 32) were unfed. The trend was similar in Layignaw-Arguba where many female *Anopheles* were unfed (48.8%; 199), and freshly-fed (47.8%; 195) (*χ* ^2^ *=* 354.5, *df* = 3, *P* < 0.001) (Table 2).

**Table 2:** Abdominal status of female *Anopheles* mosquitoes in the three study villages of Dirashe Woreda, south Ethiopia (August 2015-April 2016)

Village	Abdominal status	*n* (%)	*χ* ^2^	*P*-value
Gato	Unfed	499 (60.3)	726.2	< 0.001
	Freshly-fed	265 (32.0)		
	Half-gravid	47 (5.7)		
	Gravid	17 (2.1)		
Total		828 (100)		
Onota	Unfed	23 (71.9)	22.5	< 0.001
	Freshly-fed	7 (21.9)		
	Half-gravid	2 (6.2)		
	Gravid	0 (0)		
Total		32 (100)		
Layignaw-Arguba	Unfed	199 (48.8)	354.5	< 0.001
	Freshly-fed	195 (47.8)		
	Half-gravid	12 (2.9)		
	Gravid	2 (0.5)		
Total		408 (100)		

### CSPs rates of *Anopheles* mosquitoes

A total of 1102 CDC light traps collected *An. arabiensis* and *An. demeilloni* were tested for *Plasmodium* CSPs using ELISA (Table 3). Five *Anopheles* mosquitoes were positive for *Plasmodium* CSPs (three *An. arabiensis* in Gato and two *An. demeilloni* in Layignaw-Arguba). The overall CSP rate of *Anopheles* mosquitoes for *P. falciparum* was 0.45% (95% CI: 0.06–1.1%) (5 of 1102). *Plasmodium falciparum* CSP rate of *An. arabiensis* from Gato was 0.4% (95% CI: 0.08–1.15%) (3 of the 754) but, no *An. arabiensis* was positive for *P. vivax* CSP. In Onota, one *An. arabiensis* and 19 *An. demeilloni* were tested for CSP, and all were negative. In Layignaw-Arguba, two *An. demeilloni* were positive for *P. falciparum* CSP. Hence, the *P. falciparum* CSP rate of *An. demeilloni* was 0.64% (95% CI: 0.08–2.3%) (2 of the 311), but no *P. vivax* positive *An. demeilloni* were detected.

**Table 3:** *Plasmodium falciparum* circum-sporozoite protein (CSP) rates of *An. arabiensis* and *An. demeilloni* in the three study villages of Dirashe Woreda, south Ethiopia (August 2015-April 2016)

Villages	*An. arabiensis*		*An. demeilloni*		Overall	
	No. tested	*Pf* CSP positive (%; 95% CI)	No. tested	*Pf *CSP positive (%; 95% CI)	No. tested	*Pf* CSP positive (%; 95% CI)
Gato	754	3 (0.4; 0.08–1.15)	17	0	771	3 (0.39; 0.08–1.10)
Onota	1	0	19	0	20	0
Layignaw-Arguba	0	0	311	2 (0.64; 0.08–2.30)	311	2 (0.64; 0.08–2.30)
Total	755	3 (0.4; 0.08–1.15)	347	2 (0.57; 0.05–2.10)	1102	5 (0.45; 0.06–1.10)

*Abbreviations*: *Pf Plasmodium falciparum*, *CI* confidence interval

### EIR of *Anopheles* mosquitoes

The overall estimated *P. falciparum* EIR of *An. arabiensis* was 8.6 (95% CI: 1.7–24.8) infectious bites per person (ib/p)/nine months (Table 4). The estimated monthly *P. falciparum* EIR of *An. arabiensis* was highest in January 2016 (5.9; 95% CI: 0.7–18.7), when the density of *An. arabiensis* was also higher compared to the other months. The *P. falciparum* EIR in October was 2.9 (95% CI: 0.07–14.7) for *An. arabiensis*. The EIR was not estimated for *An. demeilloni* because the species is not so far incriminated as a malaria vector, and no conversion factor was established to estimate the human biting rate from CDC light traps.

**Table 4:** Monthly CSP rates and EIR of *An. arabiensis* and the CSP rate of *An. demeilloni* in the study villages in Dirashe Woreda, south Ethiopia (August 2015-April 2016)

Study month	Total *Anopheles* collected	*An. arabiensis*				*An. demeilloni*	
		No. tested	CSP positive *n* (%)	*Pf*EIR^a^	*Pf*EIR^b^ (95% CI)	No. tested	CSP positive *n* (%)
August 2015	5	2	0	0	0	1	0
September 2015	3	1	0	0	0	2	0
October 2015	48	32	1 (3.1)	2.95	2.95 (0.1–15.0)	8	0
November 2015	116	107	0	0	0	3	0
December 2015	178	164	0	0	0	1	0
January 2016	318	221	2 (0.9)	5.9	5.9 (0.7–19.0)	44	0
February 2016	242	2	0	0	0	186	2 (1.1)
March 2016	61	8	0	0	0	39	0
April 2016	297	0	0	0	0	63	0
Totals	1268	755	3 (0.4)	8.6	8.6 (1.7–25.0)	347	2 (0.57)

^a^Standard method: 1.91(No. ELISA positive from CDC light trap/No. ELISA tested) × (No. *An. arabiensis* collected from CDC light trap/No. of catches) × No. days in a month

^b^Alternative method: 1.91 (No. ELISA positive/No. catches) × No. days in month

*Abbreviations*: *SR* sporozoite rate, *Pf EIR* nine months *Plasmodium falciparum* entomological inoculation rate, *CSP* circum-sporozoite protein, *CI* confidence interval

### Malaria cases and *Plasmodium* species at different altitudinal transects

Of 2240 microscopy or RDT tests from malaria suspected outpatients, 405 (18.1%) were positive for *P. falciparum*, *P. vivax* or mixed infection. *Plasmodium falciparum* accounted for 34.3% (139 of the 405), whereas *P. vivax* accounted 18.3% (74 of the 405). Mixed infection of *P. falciparum* and *P. vivax* was predominant (47.4%; 192 of the 405). More mixed infections were documented by RDT test (66.1%; 127/333) in the lowland Gato (*χ* ^2^ *=* 63.68, *df =* 1, *P* < 0.001). *Plasmodium falciparum* (including the mixed infection) was the predominant species (88%; 293/333) in Gato. In Onota *P. falciparum* was also dominant species (57.5%; 23/40), whereas more *P. vivax* (59.4%; 19 of the 32) cases were reported from Layignaw-Arguba. More malaria cases were observed in Gato than in Onota and Layugnaw-Arguba (*χ* ^2^ *=* 102.2, *df =* 2, *P* < 0.001) but, the difference was not statistically significant between Onota and Layignaw-Arguba (*χ* ^2^ *=* 16.85, *df =* 2, *P =* 0.289). Although malaria cases occurred in all age groups, more malaria cases were observed in children age 5–14 years in Gato (*χ* ^2^ *=* 14.2, *df =* 3, *P* < 0.028). However, there was no statistically significant difference in number of malaria cases between Onota (*χ* ^2^ *=* 2.9, *df =* 3, *P =* 0.800) and Layignaw-Arguba (*χ* ^2^  *=*2.3, *df =* 3, *P =* 0.510) among the age groups (Table 5).

**Table 5:** *Plasmodium* species and malaria morbidity among age groups in the three study villages of Dirashe Woreda (August 2015-April 2016)

Village	Age category	Tested	Positive *n* (%)	*Pf* *n* (%)	*Pv* *n* (%)	Mixed *n* (%)	*χ* ^2^	*P-*value
Gato	< 1 year	215	8 (3.7)	1 (12.5)	2 (25.0)	5 (62.5)		
	1–4 years	460	45 (9.8)	12 (26.7)	4 (8.9)	29 (64.4)		
	5–14 years	408	117 (28.7)	26 (22.2)	13 (11.1)	78 (66.7)	14.2	0.030
	> 15 years	751	163 (21.7)	64 (39.3)	21 (12.9)	78 (47.8)		
	Total	1834	333 (18.0)	103 (31)	40 (12.0)	190 (57)		
Onota	< 1 year	10	1 (10.0)	1 (100)	0	0		
	1–4 years	29	4 (13.8)	3 (75.0)	1 (25.0)	0		
	5–14 years	38	7 (18.4)	3 (42.9)	4 (57.1)	0	2.9	0.800
	> 15 years	113	28 (24.8)	16 (57.2)	10 (35.7)	2 (7.1)		
	Total	190	40 (21.0)	23 (57.5)	15 (37.5)	2 (5.0)		
Layignaw-Arguba	< 1 year	6	1 (16.7)	0	1 (100)	0		
	1–4 years	34	2 (5.9)	0	2 (100)	0		
	5–14 years	60	5 (8.3)	2 (40)	3 (60)	0	2.3	0.510
	> 15 years	116	24 (20.7)	11 (45.8)	13 (54.2)	0		
	Total	216	32 (15)	13 (40.6)	19 (59.4)	0		
Overall total	< 1 year	231	10 (4.3)	2 (20.0)	3 (30.0)	5 (50)		
	1–4 years	523	51 (9.8)	15 (29.4)	7 (13.7)	29 (56.9)		
	5–14 years	506	129 (25.5)	31 (24.0)	20 (15.5)	78 (60.5)	21.7	0.001
	> 15 years	980	215 (22.0)	91 (42.3)	44 (20.5)	80 (37.2)		
	Total	2240	405 (18.0)	139 (34.3)	74 (18.3)	192 (47.4)		

*Abbreviations*: *Pf Plasmodium falciparum*, *Pv Plasmodium vivax*, mixed, *P falciparum* and *P. vivax*

### Seasonal pattern of malaria cases

**Fig. 3:**
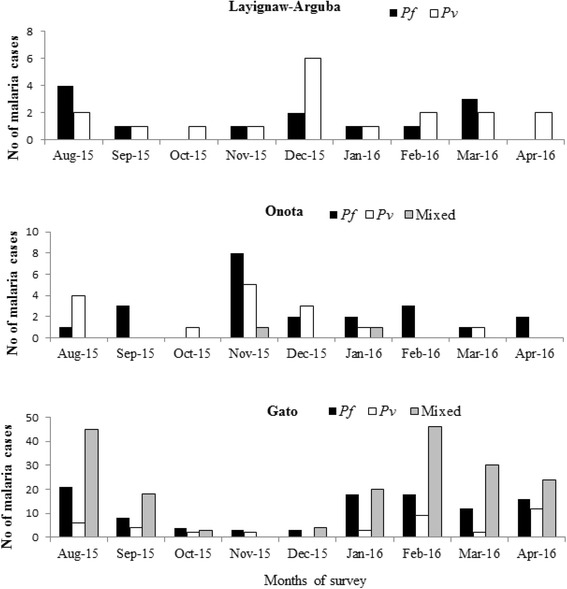
Monthly malaria cases trend in the three study villages of Dirashe Woreda, south Ethiopia (August 2015-April 2016)

There was a slight difference in malaria transmission pattern in the three study villages at different altitudinal transects. Overall in Layignaw-Arguba, an increased number of malaria cases were observed in August, December and March. In Onota more malaria cases occurred in November. More mixed infections of *P. falciparum* and *P. vivax* were reported in Gato where microscope and RDT were routinely used for malaria parasite detection. August–September 2015 and January-April 2016 were the main malaria transmission seasons in Gato (Fig. 3).

### Bednet ownership and use

Most of the households (442; 92.7%) had at least one LLIN. Of these, 160 (36.2%) had only one LLIN, and 282 (64.8%) had more than one LLIN, and the mean number was 2.1 LLINs (95% CI: 2.0–2.2). About half of the LLINs (211; 47.7%) were 2–3 years old with a mean age of 2.3 years (95% CI: 2.2–2.4) (Table 6).

**Table 6:** Bed net ownership and use in the three study villages of Dirashe Woreda, south Ethiopia (August/September 2015)

Variable / Village	Gato (*N* = 233)	Onota (*N* = 108)	Layignaw-Arguba (*N* = 136)	Total (*N* = 477)
	*n* (%)	*n* (%)	*n* (%)	*n* (%)
Bed net ownership				
Yes	228 (97.9)	101 (93.5)	113 (83.1)	442 (92.7)
No	5 (2.1)	7 (6.5)	23 (16.9)	35 (7.3)
Number of LLINs per HH				
1 net	16 (7.0)	35 (34.7)	109 (96.5)	160 (36.2)
2 nets	89 (39.0)	57 (56.4)	4 (3.5)	150 (33.9)
3 nets	65 (28.5)	8 (7.9)	0 (0)	73 (16.5)
4 and more nets	58 (25.5)	1 (1.0)	0 (0)	59 (13.4)
Source of bed net				
Government	228 (100)	101 (100)	109 (96.5)	438 (99.1)
Self-purchased	0 (0)	0 (0)	4 (3.5)	4 (0.9)
Age of bed net				
< 1 years	2 (0.9)	79 (78.2)	41 (36.3)	122 (27.6)
1–2 years	31 (13.6)	18 (17.8)	38 (33.6)	87 (19.7)
2–3 years	195 (85.5)	4 (4.0)	12 (10.6)	211 (47.7)
> 3 years	0 (0)	0 (0)	22 (19.5)	22 (5.0)
Any one slept under the bed net last night?				
Yes	198 (86.8)	72 (71.3)	73 (64.6)	343 (77.6)
No	30 (13.2)	29 (28.7)	40 (35.4)	99 (22.4)
Who slept under the bed net?				
Whole family	91 (46.0)	28 (38.9)	2 (2.7)	121 (35.3)
< 5 years (children only)	8 (4.0)	2 (2.8)	10 (13.7)	20 (5.8)
Mother and children < 5 years	93 (47.0)	39 (54.2)	50 (68.5)	182 (53.1)
Father and mother only	6 (3.0)	3 (4.1)	11 (15.1)	20 (5.8)
Bed net hanged				
Yes	193 (97.5)	72 (100)	69 (94.5)	334 (97.4)
No	5 (2.5)	0 (0)	4 (5.5)	9 (2.6)
Bed net condition				
In good condition (no holes)	197 (99.5)	70 (97.2)	57 (78.1)	324 (94.5)
Holed	1 (0.5)	2 (2.8)	7 (9.6)	10 (2.9)
Worn out/not in use	0 (0)	0 (0)	9 (12.3)	9 (2.6)

*Abbreviations*: *HH* household, *LLIN* long lasting insecticidal net

In Gato, 212 (93.0%) households owned more than one LLINs, and the rest 16 (7.0%) had one LLIN, with the mean number of 2.8 LLINs (95% CI: 2.7–2.9). In Onota 66 (65.3%) of the households owned more than one LLIN and the remaining 35 (34.7%) had only one LLIN, with the mean number of 1.8 LLIN (95% CI: 1.6–1.9). In Layignaw-Arguba, most households (109; 96.5%) were provided with only one LLIN (Table 6). Among 442 households, 343 (77.6%) reported that anyone of the family members slept under the net the night before the survey and in the rest 99 (22.4%) households, no one slept under the LLINs. More than half (53.1%; 182 of the 343) of the LLINs users were mothers and children under five years, and 121 (35.3%) were all family members. Most of the LLINs 334 (97.4%) were found hanging in the houses, and 324 (94.5%) were in good conditions (with no holes and physical damages).

There was a difference in LLINs ownership and usage at different altitudes. The overall LLIN usage was significantly higher in Gato (86.4%) compared to 71.3% in Onota and 64.6% in Layignaw-Arguba (*χ* ^2^ = 23.8, *df* = 2, *P* < 0.001).

### Indoor residual spray

**Fig. 4:**
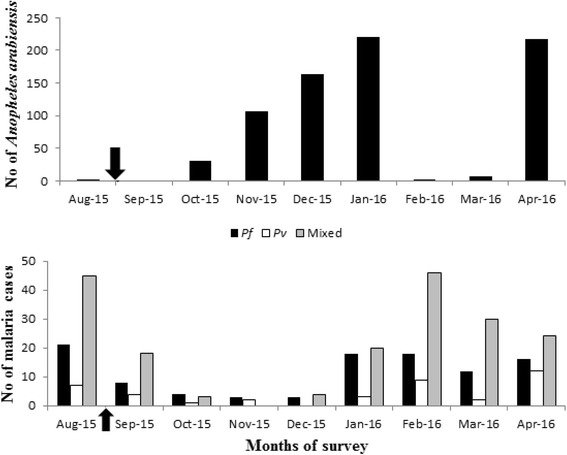
Monthly *An. arabiensis* density, malaria cases and IRS introduction time during the study period in Gato in Dirashe Woreda, south Ethiopia (August 2015-April 2016). The arrows indicate the time of indoor residual spraying

In Gato, IRS was done at the end of August and beginning of September 2015 to control malaria vectors. Of 233 surveyed houses, 150 (64.4%) were sprayed with propoxur 50% water-dispersible powder with the residual efficacy of 3–6 months at 2 g/m^2^. About 35.6% households were not sprayed. The walls of eight (5.3%) households were re-plastered after the spray. IRS was conducted when the density of *An. arabiensis* was very low, and malaria cases have already been built-up in the population (Fig. 4).

## Discussion

There was variation in species composition and infection rate of *Anopheles* mosquitoes at different altitudinal transects of Dirashe Woreda. *Anopheles arabiensis*, *An. demeilloni*, *An. cinereus*, *An. pharoensis*, *An. funestus-*group, *An. pretoriensis*, *An. christyi*, *An. ardensis* and *An. tenebrosus* were documented in the villages. *Anopheles arabiensis* was the dominant and the principal malaria vector in lowland Gato as reported in Chano Mille, south Ethiopia [18], whereas in the highland Layignaw-Arguba *An. demeilloni* was dominant and positive for *P. falciparum* CSPs. The estimated *P. falciparum* EIR of *An. arabiensis* was 8.6 infective bites per person/nine months from Gato. Malaria cases were identified in all age groups in the three villages; however, more malaria cases were documented in children age 5–14 years in Gato. *Plasmodium falciparum* was the dominant parasite species in Gato, whereas in Layignaw-Arguba *P. vivax* was dominant species.

This study has several strengths and limitations. The entomological and epidemiological data were used to assess malaria transmission patterns at different altitudinal transects. The limitation of the study was the use of secondary data from health facilities. The identification of *Anopheles* mosquito was based on the morphological characteristics which may result in misclassification as we did not use polymerase chain reaction (PCR) because of the limitation of resources and accesses.

The density of *Anopheles* mosquito was higher in Gato than Onota and Layignaw-Arguba. This finding agreed with the previous study done in the district [13]. *Anopheles demeilloni* was the dominant species next to *An. christyi* in Western Kenya highlands [19]. *Anopheles cinereus* was the second most common species in Layignaw-Arguba and Onota as it was previously documented in the highlands [3, 13]. So far these two species were not incriminated as malaria vectors in Ethiopia. However, *P. falciparum* CSP protein-positive *An. cinereus* was reported in Eritrea [20]. *Anopheles christyi* was reported from highland, and it was the second common species next to *An. arabiensis* elsewhere in Ethiopia [12]. The human-biting behaviour of *An. christyi* was reported from Akaki, in Ethiopia [3]. The study in south-central Ethiopia also identified high human blood index of *An. christyi*, *An. demeilloni* and *An. cinereus* [21]. Most *Anopheles* mosquitoes were collected in the dry season; it may be due to drying of streams and formation of small water bodies which provide suitable breading habitats for *Anopheles* mosquitoes. Similar results have been reported from highland of South-central Ethiopia [22].

*Anopheles arabiensis* from Gato and *An. demeilloni* from Layignaw-Arguba were positive for *P. falciparum* CSPs. The sporozoite rate of *An. arabiensis* for *P. falciparum* in Gato was 0.4%, which is similar to 0.32% from Chano-Mille in southern Ethiopia [23] and 0.3% in the south-central Ethiopia [21]. The *P. falciparum* CSP rate of *An. demeilloni* in Layignaw-Arguba was 0.64%. In Western Kenya, a species morphologically identified as the member of *An. demeilloni* group was positive for *P. falciparum* CSPs by ELISA and PCR [24]. *Anopheles demeilloni* has received attention because it was relatively abundant and its infectivity rate was similar to *An. funestus* group in western Kenya [24, 25]. The same species was also incriminated in Zambia, which suggests that the distribution of the species may be of regional rather than local importance in malaria transmission [26]. This is the first report in Ethiopia, and so far, no study has reported sporozoite rate of *An. demeilloni* for comparison. However, confirmation using a more sensitive method such as PCR may be advisable.

The estimated *P. falciparum* EIR of *An. arabiensis* was 8.6 infectious bites per person/nine months that were higher than expected for malaria interruption (< 1 infective bites per person per year) [27]. The nine months EIR of *An. arabiensis* was higher than the annual EIR of 3.66 infective bites per person per year from central highlands of southern Ethiopia [21]. However, it may be difficult to compare with the findings of a study in south-western Ethiopia which was 17.1 infective bites per person per year [23] since the EIR estimations of the current study were for nine months. The EIR of 8.6 in Gato is enough to sustain malaria transmission in the area, and hence the malaria control interventions should be strengthened to reduce the transmission and for future malaria elimination.

In the current study, more malaria cases were documented in the lowland Gato. It could be due to the existence of an efficient malaria vector *An. arbiensis* and the warmer climate [28]. Moreover, *P. falciparum* was the dominant malaria parasites in Gato. On the other hand, *P. vivax* was the predominant parasite in the highland Layignaw-Arguba, as reported by other studies in Ethiopia [9, 12]. It may be due to the characteristic of *P. vivax* which can develop in the vectors at a low temperature and can survive at high altitude and cool climate [29, 30]. We also documented more malaria cases in the age group 5–14 years in Gato as reported by other studies in Ethiopia [31–33]. The scenario was different in Onota and Layignaw-Arguba where more malaria cases (though not significantly different) was observed in the age groups >15 years. It could be because of the movement of people from highland to the lowland for farming and stay there sometimes and may acquire malaria parasites. When the travellers go back to homes in the highlands, they can serve as a source of infection and may result in a local malaria transmission [34].

Malaria in Layignaw-Arguba could be either indigenous, imported or both. The detection of malaria in age group <1 year who are less likely to travel to lowland and incrimination of *An. demeilloni* may imply the indigenous malaria transmission. Similarly, highlands of Ethiopia ranging from an altitude of 1500–2500 masl have experienced malaria epidemics [11]. There is also an increase in stability of malaria in highland fringes of East Africa [5, 35]. The causes for changing malaria epidemiology may be the changing climatic and ecological variables [6]. The increasing temperature can increase the digestion of blood meals taken by the mosquitoes which in turn increase human biting frequency [36] and shortens the development time of malaria parasite within the mosquito vector [6]. However, the origin of malaria at highland could be further investigated by a prospective study.

The current study revealed that 93% of the households owned at least one LLIN and 64% of the households owned more than one LLIN which is much higher than the report of Ethiopian malaria indicator survey of 2011 [37]. Despite the high ownership and utilisation of LLINs in the lowland Gato, malaria is endemic and continued as public health problem. This may be due to a shift in peak biting behaviour of *An. arabiensis* to early evening before people go to bed or the vector feeding outdoors [38]. Moreover, the percentage of children under five years in Gato who sleep under the LLINs the night preceding the survey was 51% only. This finding was lower than the report of the Ethiopia malaria indicator survey of 2011 which was 64.5% at national level, 66.1% in Southern Nations Nationalities and People’s Region state (SNNPRs) [37] and 62% in Chano Mille, south Ethiopia [39].

The percentage of households protected by IRS in Gato (IRS was applicable only in this village) was 64.4% which is lower than 80%; the recommended coverage for effective use of IRS at the community level [27]. This finding was, however, higher than the report of 2011 MIS which was 46.6% at the national level and 58.6% in SNNPRs [37]. However, the number of indoor *Anopheles* mosquitoes during the house spray was low*.* On the other hand, one month after the spray from October through January the number of indoor *Anopheles* mosquitoes was increased. This suggests that the residual efficacy of propoxur may be less than the expected duration or the sprayer may use doses less than the recommended. It may not be due to the resistance of *An. arabiensis* because they are susceptible to propoxur in most parts of the country [40]. Moreover, the spray was conducted when the number of malaria cases increased. This suggests that the spray period may not be designed based on the density of local malaria vector and malaria transmission patterns, and hence is less likely to be effective. Therefore, the policy of when to spray may need revision based on the local evidence for the effective use of IRS.

## Conclusions

There was variation in species composition and infection rate of *Anopheles* mosquitoes at different altitudinal transects. *Anopheles arabiensis* was the most dominant and the principal vector of malaria in Gato with an estimated *P. falciparum* EIR of 8.6 infective bites/ person/nine months. On the other hand, *An. demeilloni* was the predominant and positive for *P. falciparum* CSPs in Layignaw-Arguba. The incrimination of *Plasmodium*-positive *Anopheles* and presence of malaria in children under five years in Layignaw-Arguba may justify the risk of local malaria transmission in the highland of Dirashe Woreda, south Ethiopia. Hence, malaria control intervention strategies could include the highland village of the district. However, there is a need for further investigation and confirmation using more sensitive molecular techniques to consider *An. demeilloni* as a proven vector of malaria in Ethiopia.

## Abbreviations

CDC: Centres for Disease Control and Prevention
CSPs: Circum-sporozoite proteins
EIR: Entomologic inoculation rate
ELISA: Enzyme-linked immunosorbent assay
IRS: Indoor residual spraying
LLIN: Long-lasting insecticidal net
RDT: Rapid diagnostic test
